# Triangulating data sources for further learning from and about the MDSR in Ethiopia: a cross-sectional review of facility based maternal death data from EmONC assessment and MDSR system

**DOI:** 10.1186/s12884-020-02899-8

**Published:** 2020-04-09

**Authors:** Azmach Hadush, Ftalew Dagnaw, Theodros Getachew, Patricia E. Bailey, Ruth Lawley, Ana Lorena Ruano

**Affiliations:** 1 World Health Organization, Addis Ababa, Ethiopia; 2 Ethiopian Public Health Institute, Addis Ababa, Ethiopia; 3 Averting Maternal Death & Disability, Columbia University, New York, NY USA; 4 Evidence for Action, Options Consultancy Service, Addis Ababa, Ethiopia; 5 Centre for International Health, Department of Global Public Health and Primary Care, University of Bergen, Bergen, Norway

**Keywords:** Maternal death, MDSR coverage, Quality of care, Cause of death, Delay factor

## Abstract

**Background:**

Triangulating findings from MDSR with other sources can better inform maternal health programs. A national Emergency Obstetric and Newborn Care (EmONC) assessment and the Maternal Death Surveillance and Response (MDSR) system provided data to determine the coverage of MDSR implementation in health facilities, the leading causes and contributing factors to death, and the extent to which life-saving interventions were provided to deceased women.

**Methods:**

This paper is based on triangulation of findings from a descriptive analysis of secondary data extracted from the 2016 EmONC assessment and the MDSR system databases. EmONC assessment was conducted in 3804 health facilities. Data from interview of each facility leader on MDSR implementation, review of 1305 registered maternal deaths and 679 chart reviews of maternal deaths that happened form May 16, 2015 to December 15, 2016 were included from the EmONC assessment. Case summary reports of 601 reviewed maternal deaths were included from the MDSR system.

**Results:**

A maternal death review committee was established in 64% of health facilities. 5.5% of facilities had submitted at least one maternal death summary report to the national MDSR database. Postpartum hemorrhage (10–27%) and severe preeclampsia/eclampsia (10–24.1%) were the leading primary causes of maternal death. In MDSR, delay-1 factors contributed to 7–33% of maternal deaths. Delay-2, related to reaching a facility, contributed to 32% & 40% of maternal deaths in the EmONC assessment and MDSR, respectively. Similarly, delay-3 factor due to delayed transfer of mothers to appropriate level of care contributed for 29 and 22% of maternal deaths. From the EmONC data, 72% of the women who died due to severe pre-eclampsia or eclampsia were given anticonvulsants while 48% of those dying of postpartum haemorrhage received uterotonics.

**Conclusion:**

The facility level implementation coverage of MDSR was sub-optimal. Obstetric hemorrhage and severe preeclampsia or eclampsia were the leading causes of maternal death. Delayed arrival to facility (Delay 2) was the predominant contributing factor to facility-based maternal deaths. The limited EmONC provision should be the focus of quality improvement in health facilities.

## Background

Globally, an estimated 303,000 maternal deaths occurred in the year 2015. Of these, 99% came from low and middle-income countries (LMICs), with 66% of them occurring in sub-Saharan Africa [1]. Maternal death surveillance and response (MDSR) was introduced in 2013 as one of the global strategies for ending preventable maternal mortality [2, 3]. MDSR is a continuous surveillance and response cycle that involves the identification, notification, and review of all maternal deaths to provide real time actionable data for the prevention of future similar deaths. In 2015, 76% of LMICs had a national maternal death review committee in place, and 60% had a subnational maternal death review committee [4]. In the same year, most sub-Saharan African countries were implementing MDSR but in a suboptimal manner [4, 5].

Multiple factors can affect the implementation of MDSR. Lack of awareness of the purpose and principles of MDSR among stakeholders, the existence of a blame culture, insufficient number of trained staff to implement MDSR, the unavailability of guidelines and tools, lack of commitment and financial resources all contribute to the implementation of MDSR [4–13]. These barriers may prevent compliance with national and global targets for MDSR and delay the establishment of functioning MDSR committees at all health facilities [14]. Determining the facility level availability of a functional MDSR review committee helps close the knowledge gap about the implementation of MDSR at subnational level.

Several studies carried out in Ethiopia have pointed to a changing trend in the distribution of causes of maternal death [15–21]. In 2014 a systematic review revealed that the major causes of maternal death were obstructed labor/uterine rupture (36%), hemorrhage (22%), hypertensive disorders of pregnancy (19%) and sepsis/infection (13%) [22]. A review that looked at maternal deaths from 10 hospitals in Ethiopia showed that 40.3% of maternal deaths had a delay in receiving care after reaching the hospital during delivery [23]. Another facility-based maternal death review from northern Ethiopia indicated that 87.5% of maternal deaths experienced similar delays [16]. Lack of skilled health providers, incorrect diagnosis or mismanagement and shortage of appropriate medical supplies including blood for transfusion were the main reasons for delay [16, 23]. However, almost all of the studies were retrospective, from specific geographic areas of the country, and they were conducted before MDSR was introduced as a national system in 2013.

The 2016 Emergency Obstetric and Newborn Care (EmONC) Assessment was a national census of health facilities and included maternal death reviews that were conducted after the national MDSR system was introduced. In this paper the results of MDSR and the EmONC assessment are used to determine the facility level coverage of MDSR implementation, the degree to which the results from different sources were aligned on the leading causes and contributing factors to death, and the quality of care provided to the deceased women. By combining the findings from different data sources, we can better inform national programs on how to strengthen MDSR implementation, and share lessons with other countries that might consider conducting similar studies in their own context.

## Methods

### Study design and setting

A cross-sectional study design was used for this review of secondary data extracted from the EmONC assessment and MDSR system databases. The review was conducted by triangulating findings of a descriptive analysis of the extracted data. The findings from the two databases that related to the MDSR system and maternal deaths were compared. Significant agreement and disagreement were identified allowing conclusions to be drawn. The EmONC assessment included all 3804 health facilities that provide delivery services to laboring women in Ethiopia. The EmONC assessment was conducted by the Ethiopian Public Health Institute (EPHI) between May 16, 2016 and December 15, 2016 as a national cross-sectional census of health facilities. These facilities were from nine regions (Tigray, Afar, Amhara, Oromia, Somali, Benishangul-Gumuz, Sothern nations & nationalities, Gambella and Harari) and two city administrations (Addis Ababa and Dire Dawa). The EPHI also implemented routine facility based maternal death surveillance and response (MDSR) in these same facilities and maintained the MDSR database at EPHI.

### Data sources

The EmONC assessment contributed data from three different facility sources. Firstly, information on MDSR implementation was gathered by interview of facility directors or designee. Secondly, all 2015 institutional maternal deaths by cause of death as documented in the registers or logbooks were collected and thirdly, the chart reviews of the last two deceased women in the 12 months prior to the facility visit (May 16, 2016 and December 15, 2016) were reviewed. The MDSR data were derived from the facility-based maternal death review summary reports that were captured between May 16, 2015 and December 15, 2016 by the national MDSR system.

### Study variables

This study analyzed data on the implementation coverage of MDSR, the leading causes of and contributing factors to death, and the provision of quality maternal health care in health facilities of Ethiopia.

The facility based implementation coverage of MDSR was determined at national and subnational levels, and by health facility type and managing authority. The parameters assessed were the proportion of health facilities 1) with a maternal death review committee, 2) that routinely review maternal deaths, 3) with a maternal death reporting form, 4) that report maternal deaths to the next level in the system, and 5) the capture of at least one maternal death report by the national MDSR database. (Table 1) The first four of these coverage variables were captured from information collected in the interview data from the EmONC assessment. The fifth variable was found in and extracted from the National MDSR database.

**Table 1:** Percent of health facilities in Ethiopia that implement MDSR by region, facility type and managing authority, EmONC assessment and MDSR system results, 2016

	Total number of health facilities	EmONC assessment								MDSR	
		Facilities with MDSR review committee		Facilities conducted routine MD reviews		Facilities with MD reporting form		Facilities reporting reviewed MDs to next level		Facilities reporting MD review summary to national level	
	**N**	**n**	**%**	**n**	**%**	**n**	**%**	**n**	**%**	**n**	**%**
**National**	**3804**	**2435**	**64**	**1141**	**30**	**1389**	**37**	**472**	**12.4**	**211**	**5.5**
**Region**											
Tigray	255	237	93	140	55	225	88	18	7.1	17	6.7
Afar	77	19	25	15	19	7	9	8	10.4	1	1.3
Amhara	876	648	74	289	33	416	47	154	17.6	94	10.7
Oromia	1405	885	63	407	29	388	28	175	12.5	26	1.9
Somali	161	16	10	23	14	6	4	31	19.3	0	0
Benishangul-Gumuz	43	10	23	17	40	5	12	10	23.3	1	2.3
SNNP	773	510	66	193	25	251	32	59	7.6	59	7.6
Gambella	27	3	11	1	4	3	11	1	3.7	1	3.7
Harari	15	9	60	8	53	8	53	4	26.7	2	13.3
Addis Ababa	151	100	66	57	38	70	46	9	6	8	5.3
Dire Dawa	21	16	76	14	67	10	48	3	14.3	2	9.5
**Facility type**											
Referral/specialized hospitals	30	24	79	27	90	21	70	22	73.3	18	60
General hospitals	103	64	62	65	63	51	50	47	45.6	40	38.8
Primary hospitals	160	106	66	83	52	77	48	45	28.1	49	30.6
MCH specialty centers	23	3	13	7	30	3	13	1	4.3	0	0
Health centers	3459	2248	65	969	28	1233	36	356	10.3	104	3.0
MCH specialty clinics	16	0	0	1	6	3	19	0	0	0	0
Higher clinics	13	0	0	3	23	1	8	1	7.7	0	0
**Managing authority**											
Public/government	3662	2417	66	1135	31	1352	37	459	12.5	209	5.7
Private-for-profit	83	11	13	22	27	18	22	5	6	0	0
Private-not-for-profit^a^	59	20	34	19	32	19	32	8	13.6	2	3.4

*MDSR* maternal death surveillance and response, *MD* Maternal death

^a^ Includes NGO, faith-based, or mission facilities

Tables 2 and 3 show the major primary causes of death and contributing factors. The contributing factor variables assessed from both EmONC assessment and MDSR data were second delay due to delayed arrival to a health facility (Delay-2), and third delays due to delayed transfer to appropriate level of care, lack of medical equipment or supplies, absence or slowness of health worker, and incorrect diagnosis or mismanagement (Table 3). Delay 1 was not included as it was not assessed in the EmONC reviews.

**Table 2:** Percent distribution of maternal deaths by cause of death in Ethiopia, EmONC assessment and MDSR, 2016

Causes of death	EmONC 2016				MDSR	
	EmONC 2016^**a**^		Maternal death review, EmONC 2016^**b**^		MDSR (May 2015 - Dec 2016)	
	n	%	n	%	n	%
**Direct causes**	**561**	**43%**	**286**	**47%**	**511**	**85.0%**
Ruptured uterus	39	3%	23	4%	14	2.3%
Postpartum sepsis	26	2%	14	2%	53	8.8%
Severe PE/E	131	10%	61	10%	145	24.1%
Severe complications of abortion	13	1%	7	1%	11	1.8%
APH	26	2%	26	4%	42	7.0%
Obstructed/prolonged labor	52	4%	18	3%	36	6.0%
PPH/retained placenta	131	10%	111	18%	162	27.0%
Ectopic pregnancy	0	0%	2	0%	1	0.2%
Other direct causes	157	12%	26	4%	46	7.7%
**Indirect causes**	**65**	**5%**	**37**	**7%**	**58**	**9.7%**
Malaria	0	0%	0	0%	4	0.7%
HIV/AIDS related	0	0%	2	0%	2	0.3%
Anemia	26	2%	11	2%	22	3.7%
Hepatitis	0	0%	0	0%	1	0.2%
Other indirect causes	39	3%	32	5%	29	4.8%
**Unknown/unspecified causes**	**679**	**52%**	**276**	**45%**	**32**	**5.3%**
**Total**	**1305**	**100%**	**609**	**100%**	**601**	**100.0%**

*APH* antepartum haemorrhage, *PE/E* pre-eclampsia/eclampsia, *PPH* postpartum haemorrhage

^a^ Maternal deaths documented between January–December 2015 in the facility registers and records

^b^ Chart reviews of the last two maternal deaths in the last 12 months prior to data collection

**Table 3:** Percentage contribution of contributing factors, among all reviewed maternal deaths, and deaths due to major causes in Ethiopia, EmONC assessment and MDSR, 2016

Factors contributing to death (multiple responses were possible)		Data Source	All maternal deaths reviewed	PPH	APH	Obstructed or prolonged labor	Severe PE/E	Sepsis
		MDSR	(***n*** = 601)	(***n*** = 162)	(***n*** = 42)	(***n*** = 37)	(***n*** = 145)	(***n*** = 53)
		EmONC	(***n*** = 609)	(***n*** = 94)	(***n*** = 26)	(***n*** = 18)	(***n*** = 61)	(***n*** = 14)
**Delay 1**	**Traditional Practices**	MDSR	14%	14%	19%	11%	17%	21%
		EmONC	*NA*	*NA*	*NA*	*NA*	*NA*	*NA*
	**Family poverty**	MDSR	7%	6%	12%	5%	9%	9%
		EmONC	*NA*	*NA*	*NA*	*NA*	*NA*	*NA*
	**Failure to recognize the problem**	MDSR	25%	21%	19%	30%	33%	21%
		EmONC	*NA*	*NA*	*NA*	*NA*	*NA*	*NA*
	**Lack of decision to go to a health facility**	MDSR	33%	26%	43%	27%	37%	55%
		EmONC	*NA*	*NA*	*NA*	*NA*	*NA*	*NA*
	**Delayed referral from home**	MDSR	26%	27%	29%	38%	25%	34%
		EmONC	*NA*	*NA*	*NA*	*NA*	*NA*	*NA*
**Delay 2**	**Lack of roads**	MDSR	5%	7%	2%	8%	5%	2%
		EmONC	*NA*	*NA*	*NA*	*NA*	*NA*	*NA*
	**Lack of money for transport**	MDSR	2.5%	4%	5%	3%	2%	2%
		EmONC	*NA*	*NA*	*NA*	*NA*	*NA*	*NA*
	**Lack of Transportation**	MDSR	8%	14%	2%	16%	8%	2%
		EmONC	*NA*	*NA*	*NA*	*NA*	*NA*	*NA*
	**No Facility within a reasonable distance**	MDSR	4%	7%	2%	5%	3%	4%
		EmONC	*NA*	*NA*	*NA*	*NA*	*NA*	*NA*
	**Delayed arrival to a health facility**	MDSR	32%	30%	24%	49%	37%	40%
		EmONC	40%	40%	69%	67%	57%	64%
**Delay 3**	**Delayed transfer to appropriate level of care**	MDSR	22%	19%	21%	32%	22%	30%
		EmONC	29%	46%	65%	56%	36%	29%
	**Lack of medical equipment or supplies**	MDSR	16%	17%	5%	16%	19%	32%
		EmONC	17%	26%	27%	28%	16%	29%
	**Absence or slowness of health worker**	MDSR	16%	16%	10%	11%	17%	28%
		EmONC	14%	21%	12%	11%	21%	21%
	**Incorrect diagnosis or mismanagement**	MDSR	7%	7%	5%	8%	6%	13%
		EmONC	18%	21%	23%	17%	21%	7%

The causes of maternal death were used to corroborate the results of data extracted from the 1305 maternal deaths identified through facility registers during the EmONC assessment, chart reviews of 679 maternal deaths in the EmONC assessment, and reports of 601 maternal death reviews captured through the MDSR system. However, the data sources used to assess the contributing factors to maternal death were from the chart review data of the EmONC assessment and the maternal death reports of the MDSR system.

Finally, the quality of maternal health care provision was determined by the proportion of maternal deaths by cause of death who received lifesaving high impact maternal health interventions that are known as EmONC signal functions (Table 4). These variables were analyzed using data extracted from the chart reviews from the EmONC assessment; they served as the sole data source of this information.

**Table 4:** Percentage of life-saving interventions provided to women prior to their death by major causes of death, EmONC assessment, 2016^a^

	PPH	Retained Placenta	APH	Severe PE/E	Prolonged or Obstructed Labor	Postpartum Sepsis
**Treatment**	**(*n*** **= 94)**	**(*n*** **= 17)**	**(*n*** **= 26)**	**(*n*** **= 61)**	**(*n*** **= 18)**	**(*n*** **= 14)**
IV fluids	65%	69%	70%	69%	60%	87%
Plasma	5%	0%	15%	7%	0%	0%
Blood transfusion	32%	6%	30%	16%	5%	33%
Antibiotics	39%	44%	48%	61%	30%	87%
Oxytocic	48%	31%	22%	30%	25%	53%
Anticonvulsants	4%	13%	0%	72%	15%	7%
Manual removal of placenta	14%	19%	4%	7%	0%	0%
Vacuum aspiration (manual or electric)	1%	0%	0%	2%	0%	0%
Forceps	1%	0%	0%	3%	0%	0%
Vacuum extraction	0%	0%	4%	7%	0%	0%
Cesarean	11%	6%	26%	13%	25%	40%
Hysterectomy	6%	6%	11%	0%	5%	7%
Laparotomy	5%	0%	15%	0%	5%	20%
Oxygen	30%	19%	33%	54%	40%	40%

^a^ Based on chart reviews of the last two maternal deaths in the last 12 months prior to data collection

## Results

### Coverage of MDSR implementation in health facilities

Sixty-four percent of health facilities that provided delivery services in the country started MDSR implementation by establishing a maternal death review committee. However, only 30% of all health facilities conducted maternal death reviews on a regular basis, and maternal death summary report forms were available in only 37% of health facilities. It should be noted that at least 54% of the facilities reported that they had never experienced a maternal death (data not shown). Overall, 12.7% had reported at least one maternal death review summary to the next higher level in the system, and 5.5% of the facilities had one or more of their reports recorded in the national MDSR database. (Table 1).

Most of the facilities that had a report recorded in the national MDSR database were from Amhara (94), SNNP (59), Oromia (26) and the Tigray (17) regions. No facility in Somalia region had sent a maternal death report to the national MDSR database. Moreover, the major reporting facility types in the MDSR system were hospitals (107) and health centers (104). None of the maternal and child health (MCH) specialty centers or clinics recorded a report in the national database. Except for two private not-for-profit facilities, all reporting facilities were managed by the government. (Table 1).

### Causes and contributing factors to maternal death

The distribution of causes of maternal death were more or less similar in the register review and the chart review of the EmONC assessment. In the two distributions from the EmONC assessment, about half (52 and 45%, respectively) of the deceased women had no cause of death registered or it was unknown; this was true only in 5.3% of maternal deaths captured by the MDSR system. (Table 2).

The direct and indirect causes of maternal death were 43 and 5% in the register review while the remaining 52% were not categorized as either one. In the chart review 47% were categorized direct deaths, 7% indirect and 46% uncategorized. In the MDSR system, 85% of maternal deaths were due to direct causes and 9.7% died from indirect causes. Table 2 shows that all data sources had the same leading causes of death: postpartum haemorrhage or retained placenta and severe preeclampsia/eclampsia. Other direct causes of death were postpartum sepsis, antepartum haemorrhage, obstructed/prolonged labour, uterine rupture and complications of abortion. Anaemia was the most frequent indirect cause of death.

The contributing factors to maternal death were analyzed based on the reasons for the three delays to care. The EmONC assessment did not attempt to capture reasons for Delay 1 and only asked about “delayed arrival to health facility” as a Delay 2 factor since interviews with family or facility staff were not conducted. Such reasons for delay typically do not appear in patients’ records or notes and the data collectors were instructed to rely primarily on written records. “Delayed arrival from home to facility” contributed to 32% of maternal deaths reported by MDSR and 40% of maternal deaths reported by the EmONC assessment. All Delay 3 factors included in the MDSR system were assessed by the EmONC assessment. (Table 3).

The predominant Delay 3 factor occurred due to “delayed transfer of mothers to appropriate level of care,” which contributed to 22% of maternal deaths in MDSR and 29% of maternal deaths in EmONC assessment. The proportion of maternal deaths with a delay related to “lack of medical equipment or supplies” was 16% in MDSR and 17% of maternal deaths in the EmONC assessment. Delay in management due to “absence or slowness of a health worker” was reported in 16 and 14% of maternal deaths in MDSR and EmONC assessment, respectively. Incorrect diagnosis or management of mothers contributed to 7 and 18% of maternal deaths in MDSR and EmONC assessment. (Table 3).

Among the assessed contributing factors for maternal death, the maximum variation between MDSR and the EmONC assessment was 11 percentage points for “incorrect diagnosis or mismanagement.” Taking 11 points as an arbitrary maximum tolerable variation, “absence or slowness of health worker” did not show substantial differences across the major causes of death. “Delayed arrival to a health facility” showed high variation across the major causes of death except for PPH. Overall, among the five contributing factors assessed, variations less than 11 points were observed in four factors for deaths due to sepsis, three factors in PPH and severe PE/E deaths, two factors in obstructed labor deaths and one factor in deaths due to APH. (Table 3).

### EmONC service provision to deceased women

The EmONC assessment collected information on major life-saving interventions in a more systematic way than the MDSR assessment. When six of the major causes of death were examined, 60% or more of the women received IV fluids regardless of cause of death. The majority (87%) of maternal deaths due to postpartum sepsis received IV fluids and antibiotics prior to death. Moreover, 72% of deaths from severe pre-eclampsia or eclampsia (PE/E) received anticonvulsant therapy. However, less than a third of the women dying from postpartum or antepartum haemorrhage received a blood transfusion. Likewise, less than half of maternal deaths caused by PPH (48%) and retained placenta (31%) received any uterotonic drug during or after delivery. (Table 4).

Caesarean section was performed for only 25% of maternal deaths caused by obstructed labor. Hysterectomies and/or laparotomies were done in 5% of women dying of obstructed labor. Furthermore, manual removal of placenta was performed in only 19% of maternal deaths due to retained placenta. Forty percent or fewer women received oxygen with the exception of women who died of severe PE/E, in which case 54% received oxygen. (Table 4).

## Discussion

This study measured facility-level MDSR implementation by taking the maternal death review component of MDSR as its performance indicator. The outputs of maternal death review were corroborated by triangulating findings of data extracted from the MDSR system and the 2016 EmONC assessment.

The World Health Organization (WHO) recommends a phased approach for MDSR implementation and sets a target for the establishment of a maternal death review committee in every health facility [14]. However, MDSR review committee was available in only 64% of the facilities in Ethiopia. Those regions that reached more than 60% coverage were included in the initial phase of MDSR implementation while regions with less than 25% coverage were included in the last phase.

In Jigawa state of Nigeria, 41.7% of all secondary hospitals were conducting regular maternal death review (MDR) meetings after MDSR was introduced as a state system [8]. Forty-four percent of EmONC facilities in Jigawa initiated facility-based MDR but stopped after some time because of the transfer of key members of MDR committees, the lack of supportive supervision, and shortage of staff [12]. These reasons might also help explain why only 30% of health facilities in Ethiopia reported conducting routine reviews of maternal deaths. Establishing maternal death review committees does not guarantee the functionality of the committee to regularly review maternal deaths.

Although nearly a third of health facilities conducted routine maternal death reviews, only 12.4% of health facilities reported their summaries to the next level of care. The low availability of reporting forms in health facilities (37%) may contribute to this. Furthermore, the number of facilities whose reports were captured at national level was less than half of the number of facilities reporting to the next level. Other studies revealed that poor data flow from the facility to the central level was a challenge in both MDR and MDSR systems, and stemmed from a lack of knowledge and confusion among facility staff on the reporting process, high staff turnover and fear of legal repercussions [6, 7, 24].

The MDSR system had more maternal deaths with an assigned cause of death than the EmONC assessment. This could be due to methodological differences between the three systems of data collection. In the EmONC assessment, the sources of data were patients’ charts or registers, while MDSR also uses information from interviews with providers and attendants who were familiar with the circumstances surrounding the death. In addition, MDSR uses a multidisciplinary team of reviewers, unlike the review conducted by individual data collectors in the EmONC assessment. During the EmONC assessment data collection it was evident that health professionals were reluctant to report details related to maternal deaths. It is possible that they were uncomfortable assigning a cause of death, or perhaps they perceived this to be the mandate of the MDSR system.

Obstetric hemorrhage, severe pre-eclampsia and eclampsia, postpartum sepsis and obstructed labor have been shown as the leading direct causes of maternal death in countries from sub-Saharan Africa [17, 21, 25]. This is consistent with the findings in our study. The proportion of maternal deaths due to complications of abortion was less than 2% in our study and is comparable with the results of studies from Malawi and Bangladesh [24, 26].

Delay 2 factors featured prominently as contributing to more than a third of the reviewed maternal deaths in both the MDSR and EmONC assessment. This finding is confirmed by other studies from Ethiopia, Malawi and Nigeria in which Delay 2 was found to contribute to 34.7 to 59.6% of maternal deaths [16, 18, 25, 27]. Although the EmONC data failed to include specific aspects related to Delay 2, lack of transportation was found as the main contributor for Delay 2 according to the MDSR data. Studies from India and Malawi revealed that lack of transportation contributed to 16% of maternal deaths [28, 29].

The World Health Organization recommends providing parenteral oxytocin, intravenous fluid resuscitation and blood transfusions to all women with postpartum hemorrhage (PPH) and continuous bleeding [30]. The EmONC data showed that only 65% of women who died due to PPH received intravenous fluids, and 48 and 32% received oxytocin or blood, respectively. Similarly, only 72% of maternal deaths due to severe pre-eclampsia or eclampsia were provided with anticonvulsants suggesting a lack of compliance with the WHO recommendation to administer anticonvulsant therapy to treat eclampsia or prevent eclampsia in women with severe pre-eclampsia [31]. These cases reveal a critical need for greater quality of care when providing EmONC services in health facilities in Ethiopia.

This study provided critical programmatic feedback on the facility level MDSR implementation coverage, the alignment of maternal death review outputs and the provision of emergency maternal health care in the country. However, it also has limitations. A shortcoming of relying on secondary data only is that it was not possible to avoid potential sources of bias that can occur from the use of data for maternal deaths that happened during different periods of time. The register review excludes maternal deaths that occurred between January 01, 2016 and December 16, 2016, while the chart review and MDSR data excluded maternal deaths that occurred from January 01, 2015 to May 15, 2015. Methodological differences might have introduced potential bias as the chart review of EmONC used purposive sampling (the last two deaths) of maternal deaths per facility while MDSR included all maternal deaths reported to the national level. However, deaths that might have occurred in health facilities but were not reviewed or reported to the national level by the MDSR system would not appear in the MDSR database. Additionally, a large proportion of maternal deaths had missing data that were labelled as an unknown or unspecified cause in the register and chart review data of the EmONC assessment, and this might have created a potential bias in the distribution of maternal deaths by cause of death.

## Conclusion

The EmONC assessment helped determine the implementation status of MDSR. Although most health facilities in Ethiopia had MDSR committees, their overall implementation was suboptimal. Most health facilities in the country reported that they did not conduct routine maternal death reviews, and this requires further research to identify the barriers for implementation. In addition, maternal death reporting forms were available in only a few of the facilities and very few facilities reported sending the summary of a maternal death review to the next level.

Findings from the EmONC assessment corroborated the results of maternal death review outputs from MDSR by confirming the leading causes and contributing factors to facility-based maternal deaths. Obstetric hemorrhage and severe pre-eclampsia or eclampsia were the leading causes of maternal death. Delayed arrival to facility (Delay 2) was the predominant contributing factor to facility-based maternal deaths. Finally, many mothers died without receiving the evidence-based interventions that prevent maternal deaths, calling for improvements in the quality of EmONC service provision.

## Data Availability

The data that support the findings of this study are available from the Ethiopian Public Health Institute (EPHI) but data sharing restrictions apply to the availability of these data. Data are available upon reasonable request and with permission of EPHI.

## Abbreviations

n: number
NA: Not Applicable
MD: Maternal Death
MDSR: Maternal Death Surveillance and Response
MDR: Maternal death review
EmONC: Emergency Obstetric and Newborn Care
APH: Antepartum Hemorrhage
PPH: Postpartum Hemorrhage
PE/E: Pre-eclampsia/Eclampsia
